# Characterization of intrinsic and effective fitness changes caused by temporarily fixed mutations in the SARS-CoV-2 spike E484 epitope and identification of an epistatic precondition for the evolution of E484A in variant Omicron

**DOI:** 10.1186/s12985-023-02154-4

**Published:** 2023-11-08

**Authors:** Simon Schröder, Anja Richter, Talitha Veith, Jackson Emanuel, Luca Gudermann, Kirstin Friedmann, Lara M. Jeworowski, Barbara Mühlemann, Terry C. Jones, Marcel A. Müller, Victor M. Corman, Christian Drosten

**Affiliations:** 1 Institute of Virology, Charité-Universitätsmedizin Berlin, Berlin, Germany; 2 Centre for Pathogen Evolution, Department of Zoology, University of Cambridge, Downing St, CB2 3EJ Cambridge, U.K.

**Keywords:** COVID-19, SARS-CoV-2, Reverse genetics, Virus fitness, Antibody neutralization escape, Spike protein evolution, Epitope E484

## Abstract

**Background:**

Intrinsic fitness costs are likely to have guided the selection of lineage-determining mutations during emergence of variants of SARS-CoV-2. Whereas changes in receptor affinity and antibody neutralization have been thoroughly mapped for individual mutations in spike, their influence on intrinsic replicative fitness remains understudied.

**Methods:**

We analyzed mutations in immunodominant spike epitope E484 that became temporarily fixed over the pandemic. We engineered the resulting immune escape mutations E484K, -A, and -Q in recombinant SARS-CoV-2. We characterized viral replication, entry, and competitive fitness with and without immune serum from humans with defined exposure/vaccination history and hamsters monospecifically infected with the E484K variant. We additionally engineered a virus containing the Omicron signature mutations N501Y and Q498R that were predicted to epistatically enhance receptor binding.

**Results:**

Multistep growth kinetics in Vero-, Calu-3, and NCI-H1299 were identical between viruses. Synchronized entry experiments based on cold absorption and temperature shift identified only an insignificant trend toward faster entry of the E484K variant. Competitive passage experiments revealed clear replicative fitness differences. In absence of immune serum, E484A and E484Q, but not E484K, were replaced by wildtype (WT) in competition assays. In presence of immune serum, all three mutants outcompeted WT. Decreased E484A fitness levels were over-compensated for by N501Y and Q498R, identifying a putative Omicron founder background that exceeds the intrinsic and effective fitness of WT and matches that of E484K. Critically, the E484A/Q498R/N501Y mutant and E484K have equal fitness also in presence of pre-Omicron vaccinee serum, whereas the fitness gain by E484K is lost in the presence of serum raised against the E484K variant in hamsters.

**Conclusions:**

The emergence of E484A and E484Q prior to widespread population immunity may have been limited by fitness costs. In populations already exposed to the early immune escape epitope E484K, the Omicron founder background may have provided a basis for alternative immune escape evolution via E484A. Studies of major antigenic epitope changes with and without their epistatic context help reconstruct the sequential adjustments of intrinsic fitness versus neutralization escape during the evolution of major SARS-CoV-2 variants in an increasingly immune human population.

**Supplementary Information:**

The online version contains supplementary material available at 10.1186/s12985-023-02154-4.

## Introduction

Over the course of the COVID-19 pandemic, variants of SARS-CoV-2 have evolved to spread in human populations with increasing levels of immunity, showing corresponding changes in the efficiency of virus neutralization by serum-derived antibodies in cell culture [1–5]. Neutralizing antibodies primarily target the receptor-binding domain (RBD) of the viral spike (S) protein that binds to the viral entry receptor, angiotensin converting enzyme 2 (ACE2) [6–11]. The N-terminal domain (NTD) of S is an additional target of serum neutralization [12, 13]. In addition to antibody escape, changes to intrinsic viral functions may increase spread, as exemplified by virus variants D614G and variant of concern (VOC) Alpha. Those variants showed intrinsically increased fitness in the human population even in the absence of relevant changes of serum neutralization detectable in laboratory tests [14–16]. Studies of protein structure and antibody binding have generated a detailed image of the antigenic domains of S [6, 7, 9, 17, 18]. Mutagenesis studies based on early virus variants show that serum neutralization has greatest functional overlap with the activity of monoclonal antibodies targeting S amino acids 483–505, summarized as class II neutralizing antibodies [7, 17]. A particularly strong influence on immune escape capacity has been reported for amino acid substitutions at residue 484 [1, 9, 19–21]. A strong influence of E484 substitutions on overall S antigenicity was also seen in antigenic cartography studies [22].

**Fig. 1:**
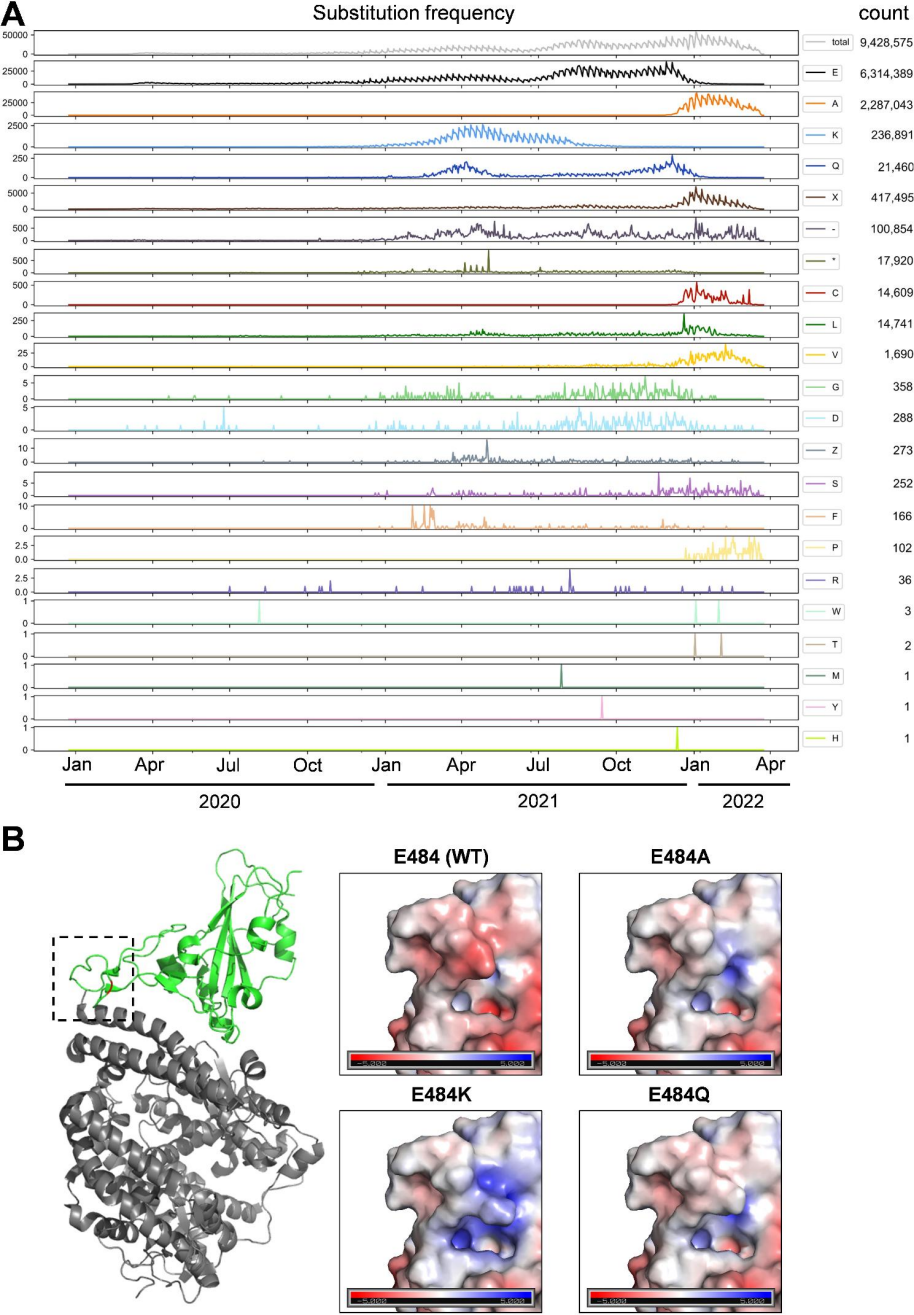
Epidemic dynamics of spike E484 variants. (**A**) Number of sequences with the indicated S:484 mutation deposited in GISAID per day (January 2020 to April 2022). (**B**) Effect of E484A, E484Q, and E484K mutations on spike protein surface charge. Left panel shows the SARS-CoV-2 spike protein (green), Protein Data Bank accession number 6LZG bound to ACE2 (grey), with residue 484 highlighted in red. The black dashed square indicates the area enlarged in the polarity display panels on right. Blue indicates positive charge. Red indicates negative charge

Several SARS-CoV-2 lineages have emerged with differing amino acid substitutions at position 484, including E484A in all stem lineages within VOC Omicron, E484Q in variant Kappa, and E484K occurring convergently in variants Eta, Theta, and the widespread VOCs Beta, Gamma, and late stage sublineages of Alpha. Consistent with their detected influence on in vitro neutralization escape, most of these lineages seem to have emerged in regions with strong recent virus transmission [23–25]. However, only three E484 substitutions detected in global SARS-CoV-2 genome sequencing efforts became temporally or permanently fixed (Fig. 1A). It is unknown to which degree these substitutions affect intrinsic fitness and what their contribution, in terms of immune escape, is towards effective fitness. As the frequency of certain E484 substitutions may be biased by linkage to other domains under selection, studies of E484 mutations in functional isolation are of interest.

Evidence for differences in ACE2 binding and serum neutralization due to isolated E484 mutations was found in RBD yeast surface display studies [26, 27], as well as by use of lenti- or rhabdoviral pseudotypes [9, 28, 29].

These techniques conveniently reflect changes in receptor affinity or cell entry but do not allow to determine virus progeny and corresponding effects on fitness over several passages. In addition, pseudotyped virus systems can involve degrees of variability not seen in the original virus, such as variability of S incorporation due to changes in RBD that are not seen in full CoV [14–16]. This uncertainty also applies to replication-competent pseudotyping systems that do not recapitulate all aspects of virion assembly, e.g., in utilizing a different budding site [30]. While these systems have been shown to adequately reflect natural CoV isolates in neutralization experiments, it is unclear whether differences in other aspects, such as glycoprotein density in virions, can reflect mutation-specific fitness changes. Experiments based on mutagenesis in full SARS-CoV-2 will more precisely reflect differential changes in fitness versus immune escape based on S mutations.

In the present study, we engineered SARS-CoV-2 variants carrying the three most frequent E484 mutations, E484A, E484Q, and E484K. We assessed viral replicative fitness against wild type (WT) in the presence or absence of immune serum, with particular focus on the founding conditions for the VOC Omicron signature E484A. To take putative epistatic changes during formation of Omicron into account, we generated an E484A variant with additional mutations H501Y + Q498R that together increase receptor binding to a high degree. Changes in Q498R cause the most dissimilar effects in WT vs. H501Y-containing Omicron background, pointing to a strong epistatic effect. Also, both mutations are entrenched in Omicron RBD, meaning that their reversion in the Omicron background causes loss of RBD affinity to a significantly greater extent than the gain by the mutations in the WT background [27, 40]. By competitive passage studies we identified differential intrinsic fitness effects undetectable by single-cycle growth or entry experiments for all E484 variants. E484A and E484Q, but not E484K, are replaced by WT in cell culture competition assays. In the presence of immune serum, all three mutants outcompete WT, suggesting that wide emergence of E484A and E484Q before establishment of population immunity may have been limited by fitness costs. We found that decreased fitness due to E484A, as present in VOC Omicron, is over-compensated to exceed the level of WT by mutations Q498R and N501Y [27, 40]. Also, the fitness gain by E484K is lost in the presence of serum raised against the E484K variant in hamsters, altogether suggesting that prior population contact with E484K-containing variants may have favored selection of E484A as soon as a conducive background was available, exemplified here by N501Y + Q498R.

## Materials and methods

### COVID-19 patient samples

Sera from triple-vaccinated individuals were available through a study on COVID-19 vaccination (EICOV/COVIM). Pre-VOC infected sera were available through a study of convalescent plasma donors, who recovered from mild to moderate COVID-19 before the emergence of any SARS-CoV-2 VOCs [45]. Sera samples were analyzed for neutralizing antibodies against wild type RBD by a surrogate virus neutralization test (cPass Neutralization Assay, Medac, Edel, Germany) according to the manufacturer’s instructions. The use of clinical samples (sera) was approved by the Institutional Review Board at Charité -Universitätsmedizin Berlin (EA2/092/20 and EA2/066/20) and is in accordance with the Berlin State Hospital Law, allowing for pseudonymized scientific analysis of routine patient data. Sera used in this study are specified in supplementary table S2.

### GISAID databank analyses

To investigate how the amino acid at position 484 of the Spike protein varied over time, we downloaded all available SARS-CoV-2 sequences from GISAID [31] on 29.03.2022, in total 9,705,558 sequences. For each sequence, we made an alignment to the Wuhan-Hu-1 reference sequence (NCBI accession NC_045512.2) using edlib [47], noted the amino acid at position 484, and the sample collection date. 276,983 sequences were discarded due to either an incomplete date or a non-human host. We analyzed and visualized the remaining 9,428,576 sequences using python 3.9.9 [53], pandas 1.2.2 [54], matplotlib 3.3.4 [55], and seaborn 0.11.1 [56].

### PyMol modeling

Wildtype SARS-CoV-2 spike protein with PDB Protein Data Bank accession number 6LZG [46] was selected to evaluate the structural effects of residue 484 mutagenesis. Images were produced using the PyMOL Molecular Graphics System (Schrödinger, LLC). Electrostatic potentials for each amino acid substitution of interest were calculated using the Adaptive Poisson-Boltzmann Solver (APBS) software suite [47].

### Reverse genetics

We employed the transformation-associated recombination (TAR) cloning method to generate infectious SARS-CoV-2 cDNA clones [50]. SARS-CoV-2 (Wuhan Hu-1 strain) subgenomic cDNA fragments cloned into pUC57 vectors were used as templates to amplify and mutagenize (NEB Site-directed mutagenesis kit) cDNA fragments required for subsequent *in yeast* cloning. We introduced spike mutation D614G into the pUC57 vector encoding subgenomic fragment 10 and E484Q, E484K, and E484A into pUC57 F9. A second round of mutagenesis PCR was performed on pUC F9 E484A to generate the pUC F9 E484A/Q498R/N501Y construct. We amplified a 5’ shortened fragment 10 (F10b) to move homologous sequences required for recombination downstream and outside of the mutated E484 codons in F9. Primers for mutagenesis and TAR fragment PCR are listed in Table 1. Assembly of amplified, purified cDNA fragments was performed as previously described [48]. Single yeast colonies were expanded and screened for correctly assembled DNA fragments using the QIAGEN Multiplex PCR kit (QIAGEN) with junction-spanning primers. PCR-positive colonies were expanded in liquid culture under histamine dropout selection (Takahara). Plasmid DNA was extracted, linearized, and subjected to T7-based in vitro RNA transcription (Thermo Fisher Scientific). Capped viral RNA was electroporated into BHK-21 cells and supernatant. One day after electroporation, culture supernatant was transferred to Vero E6 cells for virus stock production. Successful virus rescue was confirmed by SARS-CoV-2- specific RT-PCR (E gene assay [49]). Virus stocks were purified three days post-infection using Vivaspin 20 ml columns (100 kDa exclusion, Sartorius). Virus stocks were deep sequenced (NextSeq System, Illumina), confirming correct mutagenesis, the absence of second site mutations, and clonality of the furin cleavage sites in S.

**Table 1:** Oligonucleotides used in this study

Name	Sequence (5′ to 3′)	Application
F10 S:D614G F	TCTTTATCAGGGTGTTAACTGCAC	Mutagenesis PCR
F10 S:D614G R	ACAGCAACCTGGTTAGAAGTATTTG	Mutagenesis PCR
F9 S:E484Q F	CCTTGTAATGGTGTTCAAGGTT	Mutagenesis PCR
F9 S:E484A F	CCTTGTAATGGTGTTGCAGGTTTTAATTG	Mutagenesis PCR
F9 S:E484K F	CCTTGTAATGGTGTTAAGGGTTTTAATTG	Mutagenesis PCR
F9 S:E484Q/A/K R	TGTGCTACCGGCCTG	Mutagenesis PCR
F9 S:Q498R/N501Y F	GTTTCCGACCCACTTATGGTGTTG	Mutagenesis PCR
TAR F10b F	CCAACCATACAGAGTAGTAGTAC	TAR fragment
TAR F10 R	TCATGTTCAGAAATAGGACTTGTTG	TAR fragment [50]
TAR F9 F	GGAGTCACATTAATTGGAGAAGC	TAR fragment [50]
TAR F9 R	GCATCAGTAGTGTCAGCAATGTC	TAR fragment [50]
sgRNA N F	CGATCTCTTGTAGATCTGTTCTC	sgRNA N quantification
sgRNA N Prb	FAM/ CAG TAA CCA GAA TGG AGA ACG CAG /BHQ	sgRNA N quantification
sgRNA N R	CAGTATTATTGGGTAAACCTTGG	sgRNA N quantification
38F	GAAGTCAGACAAATCGCTCCAG	RT-PCR amplicon competition assay
38R	ACTAGCGCATATACCTGCACC	RT-PCR amplicon competition assay

### Cell culture

Vero E6 (ATCC CRL-1586), Calu-3 (HTB-55), NCI-H1299 (ATCC CRL-5803), and BHK-21 (ATCC CCL-10) cells were maintained at 37 °C, 5% CO2 in culture medium Dulbecco’s Modified Eagle’s Medium (DMEM, ThermoFisher Scientific) supplemented with 10% fetal bovine serum (FBS, ThermoFisher Scientific), 1% non-essential amino acids (ThermoFisher Scientific), and 1% sodium pyruvate 100 mM (NaP, ThermoFisher Scientific). Cell lines were kept at low passage and split twice a week. All cell lines tested negative for SV5 and mycoplasma contaminations.

### Virus infection and quantification assays

All infection assays were performed under BSL-3 conditions. For multicycle infection experiments, 1.5 × 10^5^ Vero E6 or NCI-H1299 cells, or 3 × 10^5^ Calu-3 cells, respectively, were seeded in a 24-well format 18–24 h prior to infection. SARS-CoV-2 stocks were diluted in OptiPRO serum-free medium (Thermo Fisher Scientific) to an MOI of 0.001 (Vero E6 and Calu-3 cells) or MOI of 0.01 (NCI-H1299 cells). For SARS-CoV-2 infections, target cells were washed once with PBS (Thermo Fisher Scientific) and incubated with 200 µl per 24-well of OptiPRO virus dilutions for 1 h at 37 °C, 5% CO2. Subsequently, virus dilutions were aspired, the cells were washed twice with PBS and supplemented with fresh culture medium. At the indicated time points post infection, culture supernatant was sampled, diluted 1:2 in 0.5% gelatine and stored at -80 °C for subsequent virus quantification by plaque titration as described below. For virus quantification by real-time PCR, culture supernatant was sampled at the indicated time points, lysed in ELB (External Lysis Buffer, Roche), and subjected to RNA extraction on the automated MagNAPure platform (Roche). Extracted viral genomic RNA was quantified by real-time PCR on a LightCycler 480 II instrument (Roche) using Taq-Man PCR probes, primers, and standards as specified in [49]. For synchronized infection assays, seeded Vero E6 and Calu-3 cells (24-well format) were placed on ice, washed once with cold PBS, and inoculated with cold virus dilutions in OptiPRO for 1 h at 4 °C. Following virus adsorption, cells were washed four times with PBS and subsequently either lysed in ELB (1 h samples) or further incubated at 37 °C for three more hours (4 h samples). ELB cell lysates were subjected to RNA extraction on an automated MagNAPure platform (Roche). Quantification of subgenomic nucleocapsid RNA (sgRNA N) was performed by real-time PCR on a LightCycler 480 Instrument II (Roche) using Taq-Man PCR probes and primers as specified in [50]. Relative sgRNA N reads were normalized to the mRNA levels housekeeping gene TBP for each sample.

### Generation of hamster sera

Six- to 10-week-old female Syrian hamsters (Mesocricetus auratus; breed RjHan:AURA) were purchased from Janvier Labs and kept in groups of 1–3 animals in individually-entilated cages (GR900; Tecniplast). Hamsters were initially infected with 1000 plaque forming units (PFU) of the respective variant and allowed to recover from infection for 21 days. On day 21 after initial infection, animals were re-infected with 1 × 106 PFU of the same variant. All infections were carried out intranasally under general anesthesia and in a total volume of 60 µl as previously described [46]. 14 days after re-infection, animals were euthanized and blood was collected for serum harvest. During the experiment, all hamsters were monitored twice daily for clinical signs of disease in compliance with a state-authority approved animal use protocol (Landesamt für Gesundheit und Soziales in Berlin, Germany, approval number 0086/20).

### Plaque titration and plaque reduction neutralization test

Plaque titrations were performed as described in [38]. In brief, seeded Vero E6 (1.5 × 10^5^ cells/well in 24-well plates) were washed with PBS, inoculated in duplicates with 1:10 serial dilutions of infectious culture supernatants for 1 h at 37 °C, and subsequently overlaid with a 1:2 mixture of Avicel (2.4 g/l, FMC BioPolymer) and double concentrated DMEM. Three dpi, the overlay was removed, cells were washed with PBS, fixed in 6% formaldehyde solution for 30 min, and then stained for 20 min in crystal violet solution. Plaque reduction neutralization tests (PRNT) were performed as described in [51]. In brief, 100 PFU of each isolate were incubated with serially diluted, heat-inactivated serum (1:40, 1:160, 1:640, 1:2560) for 1 h at 37 °C, prior to plaque titration as described above. Sera used in this study are specified in supplementary table S2.

### Competition assay

Replicative fitness was assessed as previously published for MERS-CoV [51]. In brief, Calu-3 cells seeded in 24-well format were inoculated with 10,000 PFU of recombinant virus stocks mixed in three ratios (1:1, 1:9, and 9:1 based on individual Vero-derived PFU). Infections were performed as stated above. All ratios were inoculated in biological triplicates. 24 hpi, 100 µl of infected cell culture supernatant was transferred to fresh Calu-3 cells for five subsequent passages. At each passage, viral RNA was isolated from culture supernatant using the automated MagNAPure platform (Roche). To quantify the ratio of mutant to WT or mutant to mutant in the extracted viral RNA, an 804 bp RT-PCR product over S:E484 (spanning residues 417 to 667) was amplified, using a SuperScript III One-Step RT–PCR kit (Invitrogen) and primers as indicated in Table 1. Successful amplification was verified by agarose gel electrophoresis. Amplicons were purified (ExoSAP, Invitrogen) and sent for Sanger sequencing (Microsynth GmbH). Resulting peak electropherogram heights at the codon encoding E484 and the resulting proportion of each competitor was analyzed using the web-based Chromat Quanitator (Mullins lab, University of Washington) and calculated as percentile of total peak height. Competition assays with serum pre-neutralization were performed as described above, with deviations in passaging in inoculation: Prior to p0 infection and upon each passage, 200 µl virus-containing inocula or culture supernatant were supplemented with serum in a final dilution of 1:200 and incubated for 1 h at 37 °C before infecting naïve Calu-3 cells, as described above. Hamster sera were used in final dilution 1:2000 (WT) and 1:3000 (E484K), respectively.

### Statistical analysis

**Fig. 2:**
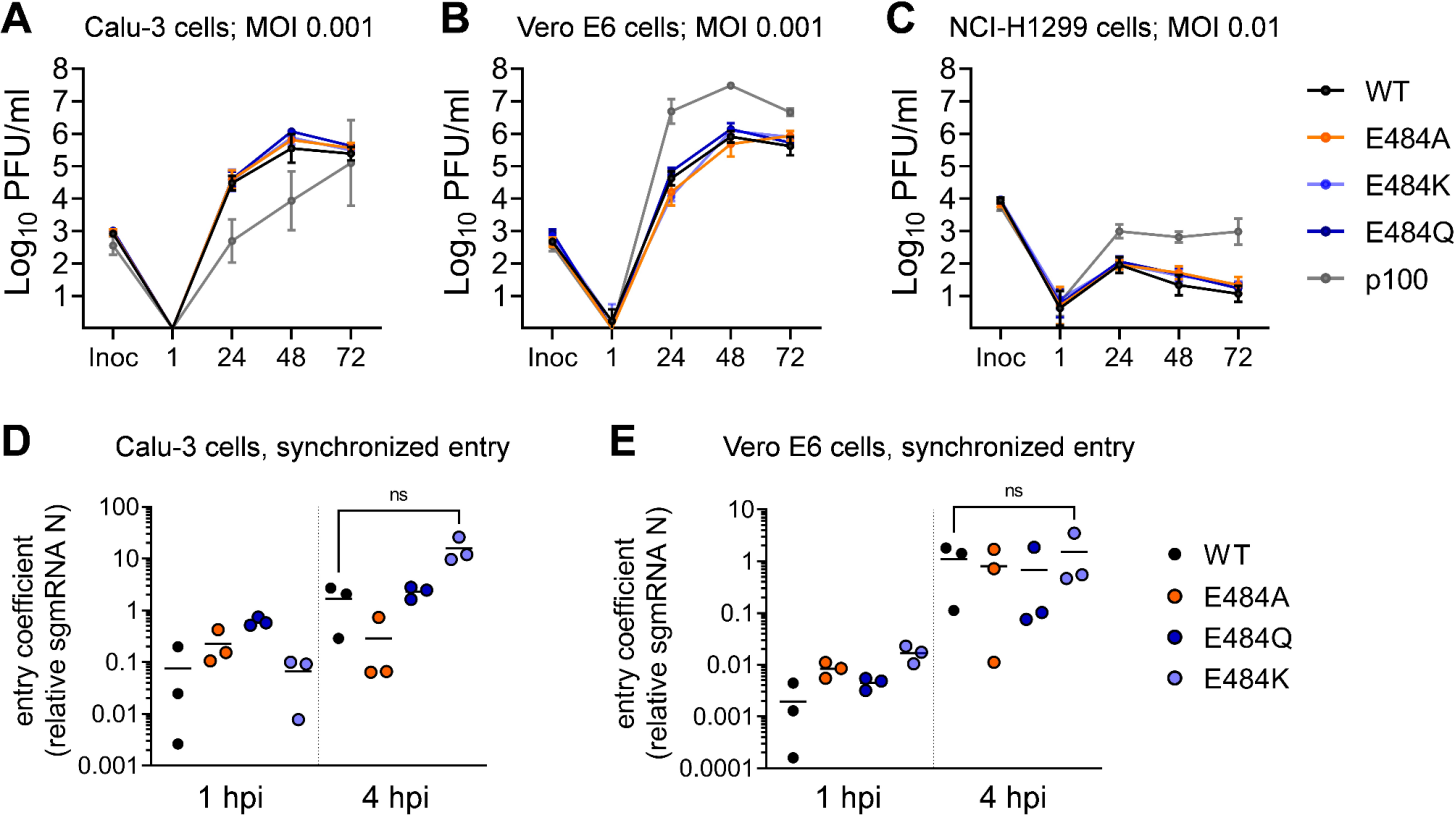
rSARS-CoV-2 spike E484 mutants display viral replication and entry kinetics similar to wildtype rSARS-CoV-2. Multicycle infection experiments in **(A)** Calu-3, **(B)** Vero E6, and **(C)** NCI-H1299 cells. Cells were infected with WT rSARS-CoV-2 or the indicated E484 mutants. As a control, we included a highly Vero E6 cell-adapted SARS-CoV-2 isolate (p100). Infectious particles sampled from culture supernatant at the indicated hpi were quantified by plaque titration. Shown are the combined data of two independent experiments, each performed in triplicate. Entry efficiency in **(D)** Calu-3 and **(E)** Vero E6 cells. Synchronized infections assays (MOI 1) were performed by virus inoculation on ice and a subsequent shift to 37 °C. Subgenomic messenger nucleocapsid RNA (sgRNA N) was quantified 1 and 4 h post infection. sgRNA N transcript levels were normalized to the housekeeping gene TBP. Significant differences in replication and entry efficiency between WT and rSARS-CoV-2 E484 mutants were determined by two-tailed Student t tests and are shown in Supplementary Table S1. MOI = multiplicity of infection; PFU = plaque-forming unit; inoc = inoculum; hpi = hours post-infection; WT = wild type

We used two-tailed Student t tests to compare differences in mean virus replication and virus cell entry efficiency between groups (GraphPad Prism v.9.3.1, supplementary Table 1). P values lower than 0.05 (P < 0.05) were considered statistically significant. In virus replication data (Fig. 2A-C) we compared the means of two experiments each performed with three independent inocula (n = 6) ± standard deviation (SD) for each group and time point. In entry data (Fig. 2D-E) we compared normalized sgRNA N amounts of a single representative experiment with three independent inocula (total n = 3) ± standard deviation (SD) for each group.

## Results

### Epidemic dynamics of spike E484 mutations in global sequencing data

Several amino acid substitutions in the SARS-CoV-2 spike protein at position E484 are implicated in immune escape [1, 9, 19–21]. To obtain an overview, we downloaded 9,705,558 SARS-CoV-2 genomes from the Global Initiative on Sharing All Influenza Data (GISAID) database [31] (https://www.gisaid.org; 29.03.2022) and plotted the number of sequences carrying spike E484 substitutions in humans uploaded to GISAID per day for every possible substitution (January 2020 to April 2022) (Fig. 1A). We identified every possible E484 substitution at least once, with a significant overrepresentation of substitutions E484A (2,287,043 sequences), E484K (236,891 sequences) and E484Q (21,460 sequences). Their temporal patterns suggest that these three mutations became fixed due to the emergence and dominance of VOCs. E484A (Fig. 1A, **orange line**) is present in all Omicron sub-lineages, and its sharp increase in frequency from the end of 2021 onward coincides with Omicron replacing previously circulating lineages [23]. This is further evident in the corresponding decline of WT E484 count (Fig. 1A, **black line**), as well as in the parallel increase of sequencing gaps (-) and undetermined amino acids (X) that were present in the early uploaded Omicron genome sequences due to primer-template mismatches and subsequent sequence misalignments [32]. E484K (Fig. 1A, **pale blue line**) frequency peaks between January 2021 and July 2021, consistent with emergence of VOCs Beta (B.1.351) and Gamma (P.1 or B.1.1.28) in southern Africa and South America, respectively [25, 33]. The increase in E484Q frequency (Fig. 1A, **dark blue line**) around April 2021 coincides with variants Kappa (B.1.617.1) and B.1.617.3 in India [34]. The decline after April 2021 is consistent with replacement by VOC Delta [34]. The second peak of E484Q frequency by the end of 2021 correlates with frequency increases of Delta sub-lineages (AY subvariants) carrying this substitution [35, 36]. All other substitutions at position 484 had low frequency throughout the observation time, with no clear patterns of emergence (Fig. 1A). The slight trend of an increase in count over time seen for all substitutions must be attributed to the overall increasing number of sequences deposited in GISAID per day (total number of sequences, Fig. 1A, **gray line**).

The apparent limitation to only three consistently detected amino acid exchanges at position 484 (E484A, E484Q, and E484K) in SARS-CoV-2 genome sequencing data is somewhat remarkable, as glutamate (E, hydrophilic, negative charge), alanine (A, hydrophobic, small), glutamine (Q, hydrophilic, uncharged) and lysine (K, hydrophilic, positive charge) have distinct biochemical properties. PyMol analysis suggests all three substitutions affect the surface charge of S (Fig. 1B).

### Recombinant SARS-CoV-2 E484 mutants show replication and entry kinetics like wildtype rSARS-CoV-2 in single and multicycle infections

We constructed recombinant SARS-CoV-2 E484 variants by reverse genetics, using the Wuhan Hu-1 genome with an additional spike D614G mutation (representative of the B.1 lineage) as an isogenic background for all mutants [50]. To assess differences in virus replication between the E484 variants, we performed multicycle infection experiments at low multiplicity of infection (MOI) in a set of cell lines providing distinct SARS-CoV-2 entry mechanisms. We used Calu-3 cells as a model for early fusion in the presence of TMPRSS2 (Fig. 2A), Vero E6 cells for endosomal virus uptake in the absence of TMPRSS2 (Fig. 2B), and NCI-H1299 cells for TMPRSS2-independent entry with very low-level ACE2 expression [37, 38]. As a control we included a Vero cell-adapted SARS-CoV-2 isolate (termed p100) that reached clearly distinct titers in all three cell lines. The differences with the p100 isolate corresponded at least in part to changes in the S1/S2 protease cleavage recognition site due to P681S and R682W exchanges present in this virus (our unpublished observations). In contrast to p100, all recombinant E484 variants replicated to infectious particle titers comparable to WT in all three cell lines (Fig. 2A-C). Also, viral RNA in cell culture supernatant did not significantly differ between any recombinant E484 variant and WT (Figure S1).

To obtain a more focused assessment of potential differences in entry efficiency, we performed single-cycle infection experiments in Calu-3 and Vero E6 cells, in which virus inoculation and attachment was performed at 4 °C, followed by a shift to 37 °C to synchronize entry of viral particles [16, 52]. We analyzed early viral subgenomic nucleocapsid (N) gene messenger RNA (sgRNA N) transcription as a correlate of early viral replication activity. In both cell lines we observed an onset of transcription at 4 h post-infection (hpi) for all E484 variants and WT, with an overall higher entry efficiency in the TMPRSS2 expressing Calu-3 cells, corresponding to observations by us and others [37, 38]. There were no significant differences in sgRNA N transcription levels at 4 hpi between E484 variants and WT (Fig. 2D-E). Only in Calu-3 cells, entry of the E484K variant seemed to exceed that of WT, but this trend was not statistically significant at a 95% confidence level (p = 0.0526 in a two-tailed Student t test; Fig. 2D-E).

### Competition assays reveal distinct effects on replicative fitness in recombinant SARS-CoV-2 E484 mutants in the absence of neutralizing human serum

**Fig. 3:**
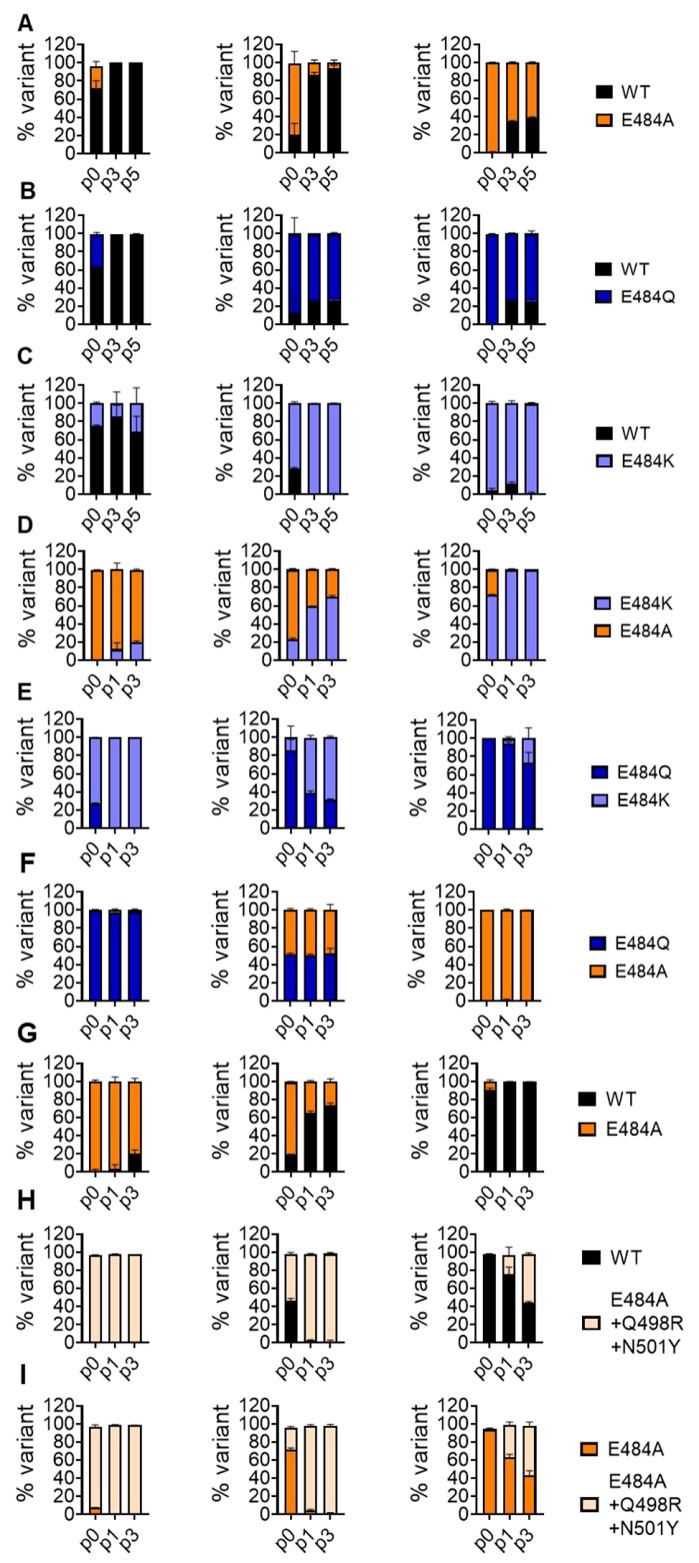
Recombinant SARS-CoV-2 S:E484 mutants show distinct replicative fitness in competition assays against wild type in the absence of neutralizing antibodies. Calu-3 cells were inoculated in triplicate with a 10,000 PFU containing mixture of two competing E484 variants or WT at two or three defined ratios (all or two of ratios 1:9, 1:1, 9:1). At 24 hpi, 100 µl of virus containing supernatant were transferred to naïve Calu-3 cultures for a total of five (**A-C**) or three (**D-I**) consecutive passages. Viral RNA was isolated from the initial inoculum (p0) and from culture supernatants at passage 1 (p1), 3 (p3), and 5 (p5) and subjected to one-step reverse transcription to generate an 800-bp PCR fragment for Sanger-sequencing. The three columns of plots show the p0 virus inoculum ratios of 1:9 (left), 1:1 (middle), and 9:1 (right) (p0 means a back-titration of the inoculum applied to the cells). Colors indicate virus mixture and identity, according to the legend on the right of each row (A-I). Peak heights in sequencing chromatograms were analyzed using the ChromatQuantitator online tool. WT = wildtype.

The small or nonexistent differences in the above experiments prompted us to refine our analysis of the effects of E484 substitutions on viral replicative fitness. We evaluated viral fitness in competition experiments, in which we inoculated Calu-3 cells with a mixture of WT and the respective E484 mutants at ratios 1:9, 1:1, and 9:1 (Fig. 3A-C). We performed five consecutive passages in naïve Calu-3 cultures, each at a 24-hour interval, and isolated viral RNA from the culture supernatant at passage 0 (p0 = inoculum back-testing), p3, and p5. To observe possible shifts in variant composition, we amplified and Sanger-sequenced an 800-bp RT-PCR fragment comprising position E484 and analyzed the resulting sequencing chromatogram. In competition experiments of WT versus E484A or E484Q, we found virus compositions at p3 and p5 shifted in favor of the WT variant, in all three ratios tested, indicative of an inferior replicative fitness of viruses carrying the E484A and E484Q substitutions (Fig. 3A and B). In contrast, competition experiments of WT versus the E484K variant showed higher relative fitness for E484K (Fig. 3C).

We next analyzed the relative fitness of each E484 variant in competition against each other. The E484K variant outcompeted the E484A and E484Q variants (Fig. 3D and E), both of which had similar fitness (Fig. 3F).

### Mutations that epistatically increase ACE2 binding compensate the fitness loss caused by E484A

The negative fitness effect of E484A contrasts with BA.1/BA.2’s increased fitness at the population level. Because several groups found increased ACE2-binding affinity for Omicron S (e.g., 39, 40), we were interested to understand epistatic context and test the contribution of mutations that may individually or synergistically increase ACE2-binding affinity [27, 40, 41]. We constructed an E484A virus that additionally contains the receptor affinity-increasing mutation N501Y present in VOC Omicron. In addition, we included Q498R present in all early Omicron lineages, suggested to contribute increased ACE-2 binding strength in an epistatic context with N501Y [27, 40]. We assessed viral replicative fitness in competition experiments against WT and the E484A variant (Fig. 3G-I). The triple mutant outcompeted the E484A mutant between 2 and 12 times as quickly as it outcompeted WT (Fig. 3H-I, Figure S2). In aggregate, the epistatic Q498R/N501Y mutations in the Omicron lineage overcompensate for the fitness loss caused by an isolated E484A mutation.

### SARS-CoV-2 E484 mutants outcompete wildtype in fitness assays in the presence of polyclonal neutralizing serum

**Fig. 4:**
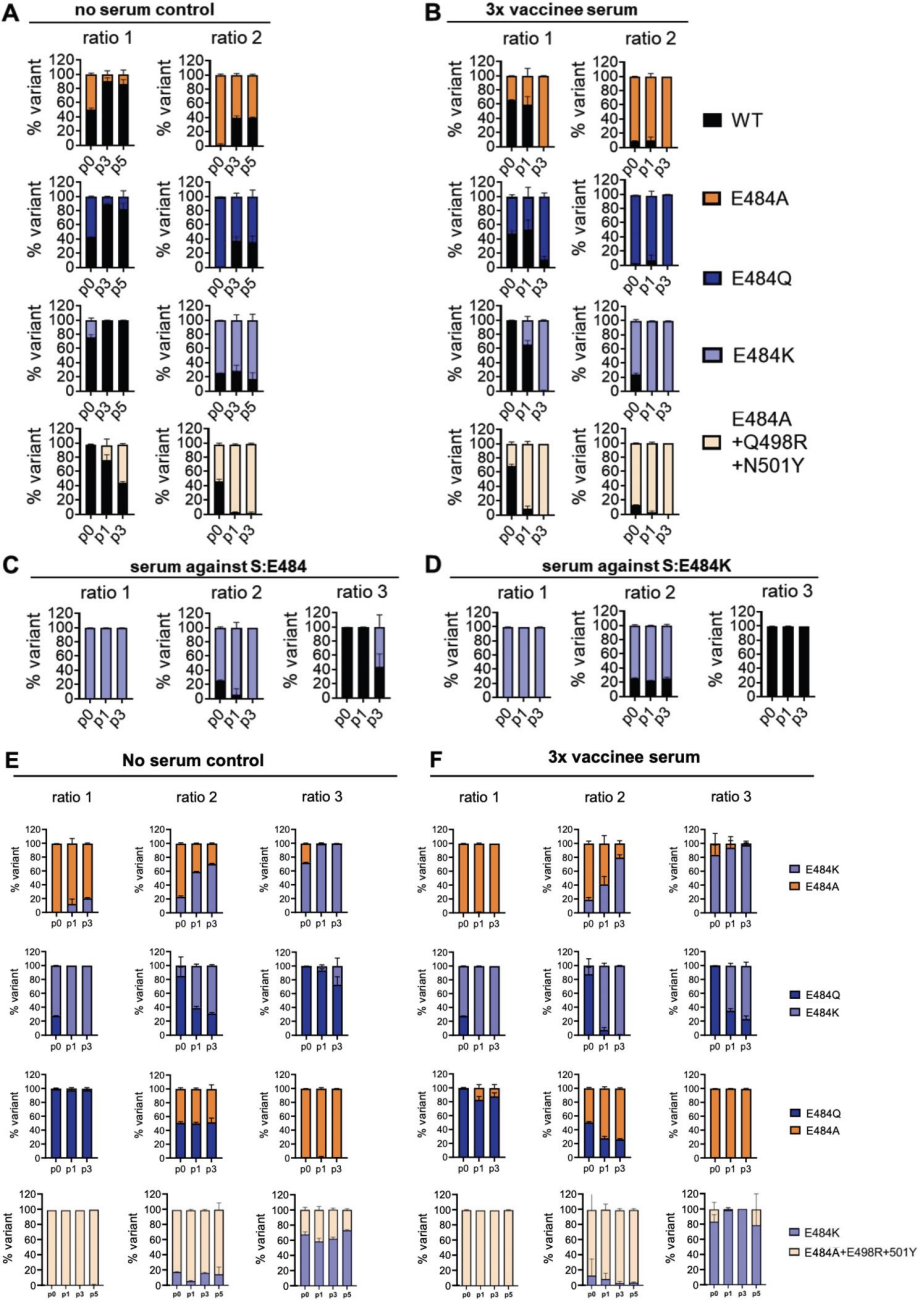
Recombinant SARS-CoV-2 S:E484 mutants show distinct replicative fitness in competition assays in the presence of neutralizing antibodies. Competition assays were performed in **A** and **E** in the absence of SARS-CoV-2 antiserum and in **B** and **F** in the presence of SARS-CoV-2 antiserum. In **C** and **D**, competition assays were performed as described above in the presence of hamster serum raised against WT (1:3000) or rSARS-CoV-2 E484K (1:2000). In **B**, virus inocula (total 10,000 PFU in three defined ratios) were pre-incubated in a 1:200 dilution of a 3x vaccinated serum for 1 h prior to infection in Calu-3 cells, or left untreated (no serum control) in **A**. Prior to infection (p0), every 24 hpi, 200 µl of virus containing supernatant were collected and incubated with a 1:200 final serum concentration, before being transferred to naïve Calu-3 cells for a total of three consecutive passages. Viral RNA was isolated from the initial inoculum (p0) and from culture supernatants at p1 and p3 (exceptionally p5) and subjected to RT-PCR yielding an 800-bp PCR fragment for Sanger-sequencing. Peak heights in sequencing chromatograms were analyzed using the ChromatQuantitator online tool. WT = wildtype.

Given the negative fitness effect of E484A and E484Q in isolation, we were interested to see whether serum neutralization might influence relative fitness among variants via neutralization escape. To estimate serum dilutions required for partial neutralization of isolated E484 variants, we conducted neutralization assays against serum from thrice-vaccinated subjects or subjects recovered from infection before the emergence of VOCs (Supplementary Figure S3). Consistent with earlier reports using S protein overexpression systems [9, 17, 42], we found all isolated E484 variants to escape serum neutralization. Titer differences between 484 variants were more pronounced in vaccinated than infected subjects (Supplementary Figure S3 A vs. B). 50% plaque reduction titers ranged between 1:160 and 1:640 in the tested sera. For competition experiments we chose one triple vaccinee serum based on availability, and used it at its 50% plaque reduction titer (1:200). In presence of this serum, all E484 variants outcompeted WT over three passages (Fig. 4A and B). The triple mutant showed the highest relative fitness under immune serum, but E484A alone was sufficient to endow the virus with greater fitness than WT in presence of serum. This suggests that selection of E484A could have initially been driven by immune escape phenomena.

To obtain an impression of the immunogenicity of escape variants, we infected hamsters twice with either WT or E484K, the variant with the highest relative fitness. E484K was then competed against WT in presence of either serum at appropriate dilutions. Under WT serum, E484K clearly outcompeted WT. Under E484K serum, it lost its increased relative fitness (Fig. 4C and D).

### SARS-CoV-2 E484 mutants show distinct fitness in the presence of polyclonal neutralizing serum

To understand selection pressures on E484 in isolation, we studied relative fitness among all pairs of E484 variants in the presence of serum from a triple-vaccinated individual. As in experiments without serum, E484K had superior fitness over E484A and E484Q (Fig. 4E). The fitness advantage of E484K over E484Q, but not over E484A, was increased by the presence of serum (Fig. 4F), arguing for a relatively lower escape capacity of E484Q. Also, in competition experiments between E484A and E484Q, the latter was inferior in the presence of serum while both had equal relative fitness in the absence of serum, supporting superior neutralization escape conferred by E484A (Fig. 4F).

To understand the basis for selection of E484A in combination with Q498R and N501Y, the triple mutant was competed against E484K. There was no discernible fitness difference over a prolonged competitive regimen of five passages in cell culture, irrespective of the presence of immune serum, suggesting that these or other epistatic ACE2-binding mutations in combination with E484A may have created an alternative to E484K for the evolution of further neutralization escape (Fig. 4E and F).

## Discussion

Here we compared the three most frequently occurring substitutions in a major antibody neutralization escape epitope (E484A, E484Q, and E484K) in the genomic context of an isogenic, recombinant SARS-CoV-2.

The study of neutralization escape mutations in a replicating SARS-CoV-2 context has certain advantages over other systems. While expressed protein affinity assays or S-pseudotyped, non-replicative virus systems can provide convenient, broad, and fast assessments of binding and entry changes, they offer no possibility to assess subtle differences in viral fitness that play a role in selection and spread of variants. Even with full CoV RBD mutants as employed here, single-cycle entry studies and multistep growth curves failed to clearly identify phenotype differences. This confirms that studies of relative fitness should use passage-competent systems that enable an experimental mimic of virus transmission chains.

Despite these advantages, the persisting cost and technical difficulty to rescue full CoV genomes limits the capacity to scan the entire RBD mutational space in full CoV genomes. For the present study we relied on preexisting data identifying E484 as a major escape epitope. Also, we focused on naturally occurring variants in circulating virus. We were particularly interested in E484A that occurred for the first time in Omicron founder lineages while E484K was previously seen in dominating immune escape variants such as Beta, Gamma, or Alpha + E484K.

Our results suggest that the selection of E484K variants may have been driven by both replicative fitness and immune escape, as E484K was able to replace WT in both the presence and the absence of neutralizing serum. The fitness-increasing component of E484K may primarily be mediated by increased ACE2 binding affinity, as several groups found positive effects of the E484K mutation on ACE2 binding using spike overexpression [26, 41, 43]. E484K was widely distributed during the Beta wave in South Africa that immunized many people during their first virus encounter without vaccination in place [23]. As genome sequencing was not well established at the time, we have to conclude by analogy that Beta must have been widespread in other parts of the African continent as well. A similar timing of infection waves caused by Gamma and Alpha + E484K in South America and Europe, respectively, suggests that relevant parts of accessible populations would have acquired E484K-derived immunity before widespread vaccination. Our results show that primary E484K antigen contact in hamsters results in mutation-specific immunity that abolishes the fitness effect of E484K. A growing number of E484K-exposed individuals along with the onset of vaccination programs might therefore have caused population-level immune selection against E and K prior to Omicron emergence.

According to our data, both E484A and E484Q in isolation reduce fitness, which corresponds to their limited emergence prior to the establishment of widespread population immunity. However, in the presence of antibody neutralization, E484A affords slightly better immune escape than E484Q, suggesting that selection during VOC Omicron emergence might have favored E484A variants over E484Q.

The reduction of ACE2 binding by E484A has been associated with a smaller ACE2 binding interface [26, 39], arguing for decreased ACE2 affinity as the cause of fitness loss by this mutation. The evolution of an immune escape trait that decreases intrinsic fitness may be facilitated by a conducive epistatic background that compensates for affinity loss. The H501Y mutation present in all Omicron lineages is a well-characterized affinity-increasing mutation. Further epistatic increase of receptor affinity by acquisition of Q498R on top of H501Y was predicted even before the emergence of Omicron by in vitro evolution studies based on yeast surface-expressed RBD and structural biology inferences [27]. After the emergence of Omicron, position 489 was found by another group to undergo the strongest epistatic change of all RBD positions in Omicron versus wildtype, based on yeast surface-expressed RBD mutation scanning and in vitro affinity measurements [40]. Epistatic dependence on H501Y is suggested by the fact that changes in position 498 are most dissimilar in their effects on receptor binding in H501Y-containing backgrounds (Omicron BA.1, Omicron BA.2, Alpha, Beta) as compared to wildtype background [40]. Also, in Omicron, these two positions undergo epistatic entrenchment, based on the observation that their reversion in the Omicron background causes reduction of RBD affinity to a significantly greater extent than the gain by the mutations in the WT background. These data suggested H501Y + Q498R to be a likely epistatic background during foundation of Omicron with additional acquisition of E484A. The here-tested E484A + H501Y + Q498R triple mutant not only matched E484K in terms of fitness, but also reached the same or higher level of neutralization escape. The E484A + H501Y + Q498R combination might thus have opened an evolutionary opportunity for the acquisition of further immune escape mutations in a putative Omicron precursor.

It is unclear whether earlier immune escape variants also involved the evolution of a conducive background, as the simultaneous acquisition of epistatically-active mutations is statistically unlikely. Interestingly, in vivo experiments found no overall increased fitness of the Beta VOC (encoding not only E484K, but also K417N and N501Y) [44], suggesting that fitness-increasing effects of E484K may be abolished by other mutations in VOC Beta. A likely candidate is the spike K417N mutation, which has been shown to negatively affect ACE2 binding [41]. Also, it is interesting to note that E484Q which we found inferior to E484A was not selected in experimental evolution studies based on a H501Y background [27]. E484Q was primarily detected in Kappa and VOC Delta sublineages [35, 36] and may hence have evolved in variants with intrinsically increased fitness, possibly mediated by L452R [45, 46].

Our study has several limitations. First, in absence of data on early representatives of Omicron stem lineages, we can only infer from *in-vitro* data that Q498R was the most likely epistatic mutation to occur with H501Y and allow onward immune escape evolution by compensation of fitness costs. Other mutations in sum may have similar effects, albeit their combined acquisition is much less likely. Also, our *in-vitro* experiments are conducted on cultured cells that may not faithfully reflect all attachment and entry factors determining virus fitness *in vivo.* In particular, the use of cell lines expressing a high level of ACE2 may not entirely reflect the conditions in the respiratory tract where lower ACE2 expression prevails in most tissues. Other factors, including mucus, saliva, and other components of the mucosal fluid milieu cannot be appropriately studied in the deployed models. Furthermore, we have not used a panel of different human sera for competition experiments as the effort associated with competitive serial passaging is high. Also, present cell culture models cannot recapitulate possible contributions of E484 substitutions to the evasion of cellular immunity and fitness effects thereof. In sum, this study demonstrates that immune escape assays that also consider the fitness dimension can further our understanding of antigenic evolution-based laboratory models.

## Conclusions

The present study separates consequences on fitness from those on neutralization escape for fixed mutations in a major SARS-CoV-2 immune escape epitope. The emergence of E484A and E484Q prior to widespread population immunity may have been limited by fitness costs. In populations already exposed to the early immune escape mutation E484K, the combined Omicron mutations H501Y and Q498R may have provided an epistatic context enabling the selection of E484A as an alternative immune escape trait despite its fitness cost in isolation.

### Electronic supplementary material

Below is the link to the electronic supplementary material.

Supplementary Material 1
