# Supplementary material for: Characterization of intrinsic and effective fitness changes caused by temporarily fixed mutations in the SARS-CoV-2 spike E484 epitope and identification of an epistatic precondition for the evolution of E484A in variant Omicron
Source: Virol J. 2023 Nov 8;20:257. doi: 10.1186/s12985-023-02154-4 (PMC10633978; doi:10.1186/s12985-023-02154-4)
Supplement: Supplementary file 1 — Supplementary Material 1 [file 12985_2023_2154_MOESM1_ESM.pdf]

Supplementary data to

Characterization of intrinsic and effective fitness changes caused by temporarily fixed mutations in the SARS-CoV-2 spike E484 epitope and identification of an epistatic precondition for the evolution of E484A in variant Omicron

Authors:

Simon Schroeder[1], Anja Richter[1], Talitha Veith[1], Jackson Emanuel[1], Luca Gudermann[1], Kirstin Friedmann[1], Lara M. Jeworowski[1], Barbara Mühlemann[1], Terry C. Jones[1,2], Marcel A. Müller[1], Victor M. Corman[1], Christian Drosten[1,#]

Affiliations:

[1]Institute of Virology, Charité-Universitätsmedizin Berlin, Berlin, Germany

[2]Centre for Pathogen Evolution, Department of Zoology, University of Cambridge, Downing St., Cambridge, CB2 3EJ, U.K.

#Corresponding author

e-mail: christian.drosten@charite.de

Supplementary data

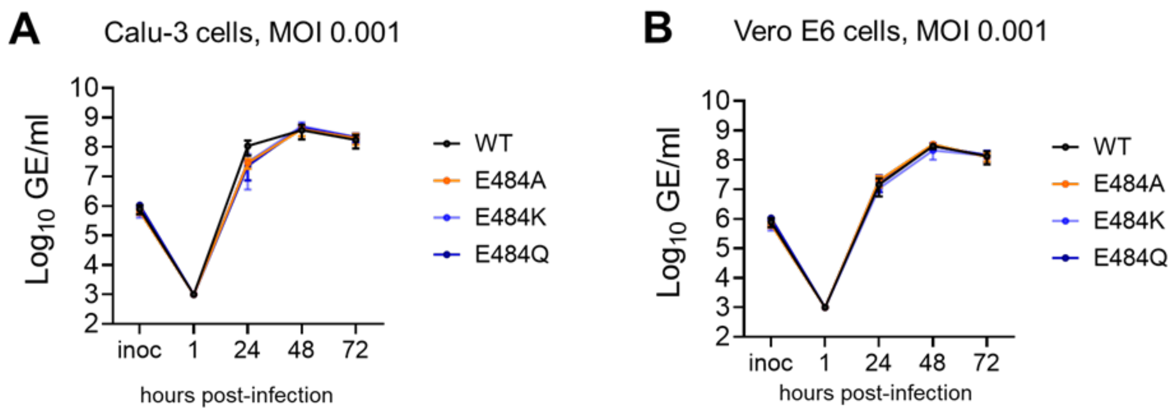

Figure S1: rSARS-CoV-2 S:E484 mutants display highly similar replication kinetics in Calu-3 and Vero E6 cells. Multicycle infection (MOI 0.001) in **A)** Calu-3 cells and **B)** Vero E6 cells with the indicated rSARS-CoV-2 mutants were performed. At the indicated hpi, viral RNA was extracted from the culture supernatant and quantified using real-time quantitative PCR (E assay). Shown are the combined data of two independent experiments performed in triplicates (n = 6). MOI = multiplicity of infection; WT = wild type; GE = genome equivalents

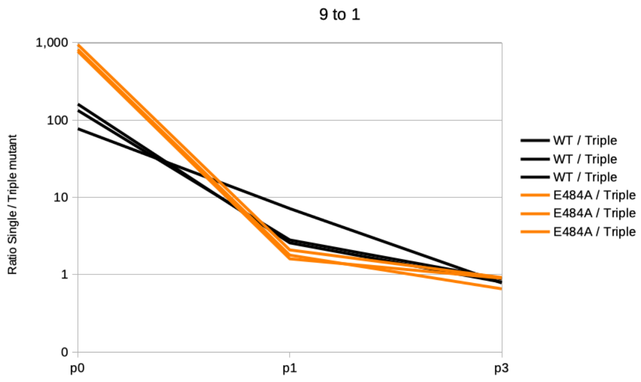

Figure S2: Comparison of WT and E484A versus triple mutant growth dynamic in competition assay. Data from the 9:1 (triple-mutant initially in minority) infection of the competition experiment without serum, taken from the rightmost column of Figure 3H and 3I. For each timepoint (p0, p1, p3), the ratio of single mutant (WT or E484A) fraction to triple mutant (E484A, Q498R, N501Y) fraction, was computed. Each line connects the ratios of one biological repeat out of three for each of the two competitions. The steeper decline of the orange lines shows the more rapid reduction over time of the fraction of E484A mutant in the presence of the triple mutant than the reduction of WT (black lines) in the presence of the triple mutant. A normalization (not shown) of these ratios relative to the mean ratio of WT versus triple mutant ratio, indicates the triple mutant out-competing the E484A mutant between 2 and 12 times as quickly as it out-competed WT.

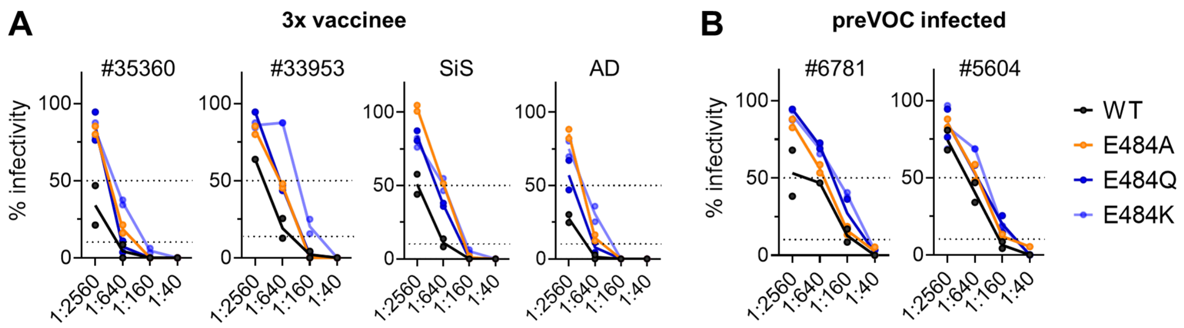

Figure S3: 50 PFU of each variant were incubated in duplicates with the indicated serial dilutions of SARS-CoV-2 antisera in culture medium, or culture medium alone (no serum control), for 1 hour prior to infection of Vero E6 cells. At 3 dpi, cells were fixed, stained, and plaques were counted. Shown here is the average reduction of plaques over no serum control, i.e., reduction in infectivity ± SD. Dashed lines show 50 and 10% infectivity, respectively. PRNTs were performed with antisera of **A)** triple-vaccinated donors (pre-VOC vaccinated), **B)** pre-VOC infected donors (B.1 infected)

Table S1: P-values for Figure 2. Statistical differences in replication (PFU/ml) between groups were analyzed by two-tailed Student *t* tests (GraphPad Prism v.9.3.1).

Cell line	Sample	Comparision	P-value
Calu-3 (Fig 2A)	24 hpi	WT vs 484A	ns; 0.4777
		WT vs 484Q	ns; 0.3828
		WT vs. 484K	ns; 0.6472
	48 hpi	WT vs 484A	ns; 0.1871
		WT vs 484Q	ns; 0.0578
		WT vs. 484K	ns; 0.1296
	72 hpi	WT vs 484A	ns; 0.1283
		WT vs 484Q	ns; 0.0625
		WT vs. 484K	ns; 0.2802
Vero E6 (Fig 2B)	24 hpi	WT vs 484A	ns; 0.0515
		WT vs 484Q	ns; 0.0728
		WT vs. 484K	ns; 0.0533
	48 hpi	WT vs 484A	ns; 0.2283
		WT vs 484Q	ns; 0.0539
		WT vs. 484K	*; 0.0392
	72 hpi	WT vs 484A	ns; 0.0774
		WT vs 484Q	ns; 0.4432
		WT vs. 484K	ns; 0.0921
H1299 (Fig 2C)	24 hpi	WT vs 484A	ns; 0.9563
		WT vs 484Q	ns; 0.4050
		WT vs. 484K	ns; 0.7466
	48 hpi	WT vs 484A	*; 0.0330
		WT vs 484Q	*; 0.0492
		WT vs. 484K	ns; 0.0929
	72 hpi	WT vs 484A	ns; 0.0913
		WT vs 484Q	ns; 0.1866
		WT vs. 484K	*; 0.0243

Table S2: Information on sera used

Serum	Category	Date of infection or vaccination	Date of sample collection	collection post infection or vaccination (days)	cPass WT
**35360**	3x vaccinee	30.11.2021	04.01.2022	35	95.71
**33953**	3x vaccinee	24.10.2021	23.11.2021	30	95.81
**SiS**	3x vaccinee	02.12.2021	02.03.2022	90	96.18
**AD**	3x vaccinee	03.01.2021	02.03.2022	59	96.15
**6781**	preVOC infection	17.03.2020	07.07.2020	112	93.20
**5604**	preVOC infection	09.03.2020	06.05.2020	58	90.50

Table S3: Oligonucleotides used in this study

Name	Sequence (5´ to 3´)	Application
F10 S:D614G F	TCTTTATCAGGGTGTTAACTGCAC	Mutagenesis PCR
F10 S:D614G R	ACAGCAACCTGGTTAGAAGTATTTG	Mutagenesis PCR
F9 S:E484Q F	CCTTGTAATGGTGTTCAAGGTT	Mutagenesis PCR
F9 S:E484A F	CCTTGTAATGGTGTTGCAGGTTTTAATTG	Mutagenesis PCR
F9 S:E484K F	CCTTGTAATGGTGTTAAGGGTTTTAATTG	Mutagenesis PCR
F9 S:E484Q/A/K R	TGTGCTACCGGCCTG	Mutagenesis PCR
F9 S:Q498R/N501Y F	GTTTCCGACCCACTTATGGTGTTG	Mutagenesis PCR
TAR F10b F	CCAACCATACAGAGTAGTAGTAC	TAR fragment
TAR F10 R	TCATGTTCAGAAATAGGACTTGTTG	TAR fragment (48)
TAR F9 F	GGAGTCACATTAATTGGAGAAGC	TAR fragment (48)
TAR F9 R	GCATCAGTAGTGTCAGCAATGTC	TAR fragment (48)
sgRNA N F	CGATCTCTTGTAGATCTGTTCTC	sgRNA N quantification
sgRNA N Prb	FAM/ CAG TAA CCA GAA TGG AGA ACG CAG /BHQ	sgRNA N quantification
sgRNA N R	CAGTATTATTGGGTAAACCTTGG	sgRNA N quantification
38F	GAAGTCAGACAAATCGCTCCAG	RT-PCR amplicon competition assay
38R	ACTAGCGCATATACCTGCACC	RT-PCR amplicon competition assay
